# ROKU: a novel method for identification of tissue-specific genes

**DOI:** 10.1186/1471-2105-7-294

**Published:** 2006-06-12

**Authors:** Koji Kadota, Jiazhen Ye, Yuji Nakai, Tohru Terada, Kentaro Shimizu

**Affiliations:** 1 Graduate School of Agricultural and Life Sciences, The University of Tokyo, 1-1-1 Yayoi, Bunkyo-ku, Tokyo 113-8657, Japan

## Abstract

**Background:**

One of the important goals of microarray research is the identification of genes whose expression is considerably higher or lower in some tissues than in others. We would like to have ways of identifying such tissue-specific genes.

**Results:**

We describe a method, ROKU, which selects tissue-specific patterns from gene expression data for many tissues and thousands of genes. ROKU ranks genes according to their overall tissue specificity using Shannon entropy and detects tissues specific to each gene if any exist using an outlier detection method. We evaluated the capacity for the detection of various specific expression patterns using synthetic and real data. We observed that ROKU was superior to a conventional entropy-based method in its ability to rank genes according to overall tissue specificity and to detect genes whose expression pattern are specific only to objective tissues.

**Conclusion:**

ROKU is useful for the detection of various tissue-specific expression patterns. The framework is also directly applicable to the selection of diagnostic markers for molecular classification of multiple classes.

## Background

A major challenge of microarray analysis is to detect genes whose expression in a single or small number of tissues is significantly different than in other tissues. Accurate identification of such tissue-specific genes can allow researchers to deduce the function of their tissues and organs at the molecular level [1].

Several methods have been used for this purpose [1-5]. Of these, Schug et al. [4] demonstrated the effectiveness of using Shannon information theoretic entropy for ranking genes according to their tissue-specificity, from restricted (tissue-specific) expression to average (ubiquitous/housekeeping) expression. However, there is also a severe disadvantage. The entropy does not explain to which tissue a gene is tissue-specific, only measuring the degree of overall tissue specificity of the gene. Hence further analysis to identify specific tissues is needed. Although Schug et al. [4] proposed a new statistic (*Q*) based on entropy to estimate the degree of a gene's specificity on a particular tissue, the issue of redundancies remains where top-ranked genes as specific to tissue *A *are also top-ranked as specific to tissue *B*. We assert such genes are not specific to *A *or *B*, but rather are genes specific to both *A *and *B*. For example, we observed that two of the top five probesets specific to liver were also found in the top five probesets specific to gall bladder [4]. The issue of such redundancies is a concern with any ranking-based method, such as pattern-matching [2], when the number of interrogated tissues increases. Methods of identifying genes specific only to objective tissues are needed.

Unlike ranking-based methods, methods based on outlier detection are free from the issue of redundancies because they identify tissues corresponding to both over- and under-expressed outliers for each gene [3,5]. Therefore, these methods can treat equally various types of tissue-specific genes: (1) 'up-type' genes selectively over-expressed in a single or small number of tissues compared to the others, (2) 'down-type' genes selectively under-expressed, and (3) 'mixed-type' genes selectively over- and under-expressed in some tissues. Although the mixed-type is possible, the first two types (up-type and down-type) of expression patterns are particularly important because those genes may be associated with fundamental biological phenomena and may contain particular tissue-specific diagnostic markers. Using outlier-detection-based methods, however, ranking genes according to their degree of overall tissue-specificity is difficult.

This complementary relationship between ranking-based and outlier-based methods led us to develop a combined approach, ROKU. ROKU analyzes any type of tissue-specific genes (up-, down-, and mixed-type) in two steps. First, it ranks genes according to overall tissue-specificity using Shannon entropy, and second, for each gene, it identifies specific tissues whose observations are regarded as outliers using a method of Kadota et al. [3]. We applied the method to both synthetic and real gene expression data and demonstrated its utility by comparison with other methods.

## Results and discussion

### Definition of tissue-specific genes

**Figure 1:**
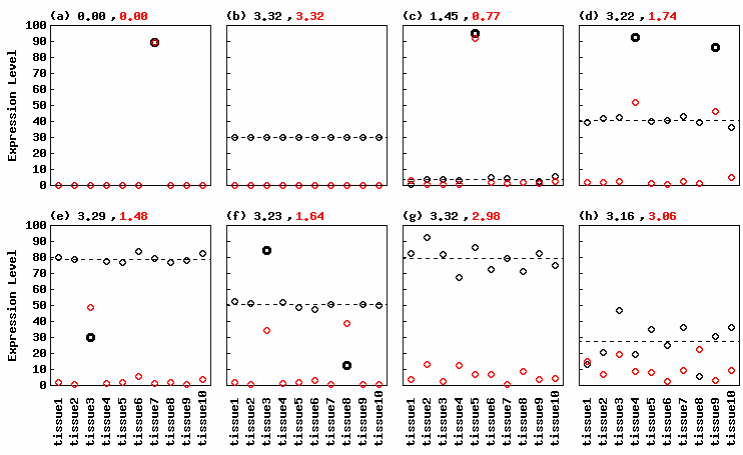
**Shannon entropy calculation for various tissue-specific expression patterns**. Synthetic expression patterns are shown. Original expression data represented by black circles are processed by equation 2 using Tukey biweights (dashed line). Processed data are represented by red circles. Specific expression observations detected to be outliers by [3] are highlighted. Numbers in black and red indicate Shannon entropy scores for the original and the processed data, respectively. Shannon entropy can range from (a) 0 to (b) 3.32 in this case (logarithm to the base 2 of 10). Expression patterns such as (a) and (c) are defined as tissue-specific genes in a narrow sense. Tissue-specific genes in a broad sense include various expression patterns such as (a, c, d) up-type, (e) down-type, and (f) mixed-type. By virtue of data processing, ROKU can detect tissue-specific genes in a broad sense. Meanwhile, ROKU gives relatively high scores (close to 3.32) for non-specific gene expression patterns such as (g) and (h).

We first show typical examples of various types of gene expression patterns. We here divided tissue-specific genes into two levels, a narrow sense and a broad sense. Genes over-expressed in a small number of tissues but unexpressed or slightly expressed in others, such as those shown in Figs. 1a and 1c, are defined as tissue-specific genes in a narrow sense, while genes over- and/or under-expressed in a small number of tissues compared to other tissues are defined as tissue-specific in a broad sense (the latter group includes the former). We focused here on the latter case and wanted to identify such expression patterns (see black scatter plots in Figs. 1d–f). We use two terms ("genes" and "probesets") interchangeably throughout this paper.

### Data processing and its effect on Shannon entropy calculation

When one gene vector ***x ***= (*x*_*1*_, *x*_*2*_, ..., *x*_*N*_) is given, the entropy *H*(***x***) can be calculated by equation 1 (See Methods). The range of *H *is from 0 whose gene expression is perfectly restricted in a single tissue (Fig. 1a) to log_2_(*N*) whose gene expression pattern is flat in all the interrogated tissues (Fig. 1b). We therefore rely on the low entropy score for the identification of tissue-specific genes. The black scatter plots in Fig. 1 are synthetic expression observations for *N *tissues (i.e., *N *= 10 in this case). The entropy *H *for each gene vector ***x ***is given by the number in black above the figures. Clearly, direct calculation of the entropy for raw gene vector ***x ***works well only for detecting tissue-specific genes in a narrow sense (Figs. 1a and 1c) but not for those in a broad sense (Figs. 1d–f). The *H *scores (3.22, 3.29, 3.23 for Figs. 1d–f, respectively) of tissue-specific genes in a broad sense are close to the maximum value (log_2_10 = 3.32) and cannot identify those genes as 'tissue-specific'.

To detect tissue-specific genes in a broad sense, we introduce a simple method that processes a given gene vector ***x ***and makes a new vector ***x***'. Data processing is done by subtracting the one-step Tukey biweight and by taking the absolute value of equation 2 (see Methods). The Tukey biweight yields a robust weighted mean able to resist 50% of outliers [6]. The scatter plots of processed vectors are shown in red in Fig. 1. The entropy scores, *H*(***x***'), for the processed vectors to obvious tissue-specific genes in a broad sense (Figs. 1d–f) are considerably lower than those for ***x***. This is because the relative expression levels for specific tissues (highlighted tissues) become high after data processing. For example, the value (0.04) for tissue 3 in Fig. 1e becomes 0.75 after data processing. Since the base-line value is 0.1 (1/*N*, *N *= 10) in this case, such high values decisively contribute low entropy to the gene expression pattern. Also, entropy scores, *H*(***x***') and *H*(***x***), to non-specific (or randomly expressed) genes are quite similar and close to the maximum (3.32) (Figs. 1g and 1h). These results demonstrate the adequacy for our strategy for detecting tissue-specific genes in a broad sense at least on typical/hypothetical expression data.

### Analysis of real data

To further investigate the validity of our method (ROKU), we applied the method to a public gene expression matrix consisting of 36 normal human tissues and 22,283 probesets [5]. Briefly, ROKU (1) processes each probeset expression vector and makes a processed vector ***x***', (2) calculates the entropy *H*(***x***'), and (3) assigns specific tissues to each probeset whose observations are detected to be 'outliers' (see Methods). We compared the performance of ROKU to that of Schug's method, which directly uses the original/non-processed vector ***x ***for measuring the entropy *H*(***x***) [4]. The two entropy scores (*H*(***x***') and *H*(***x***)) for all probesets are available in the additional file [see Additional file 1].

To compare the agreement of top-ranked probesets between ROKU and Schug's method we analyzed the percentage of common probesets in a top-ranked set of ~22,283 probesets. About 80% of ~3,000 top-ranked probesets are common, indicating that ROKU does not change the rank of probesets drastically (data not shown). One way to compare the effect of the data processing used in ROKU to that used in Schug's method is to sort probesets in order of increasing magnitude by the difference between the two entropy scores (*H*(***x***') - *H*(***x***)) calculated by the two methods. Since ROKU outputs relatively low entropy to each probeset compared to Schug's method as a whole [see Additional file 1], the average value of (*H*(***x***') - *H*(***x***)) tends to be negative: -0.425 (4.314 for ROKU; 4.739 for Schug's method).

**Figure 2:**
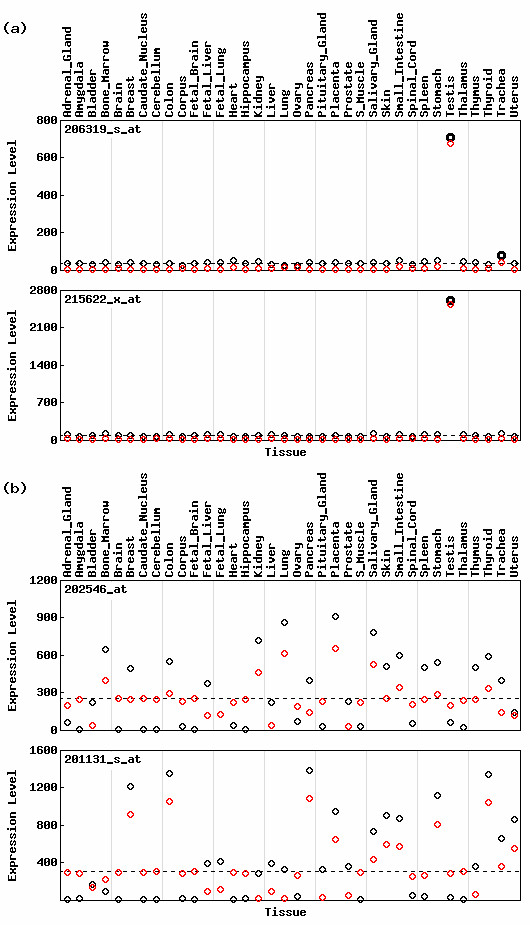
**Expression patterns of probesets listed in Table 1**. Expression patterns of probesets with the two (a) lowest- and (b) highest (*H*(***x'***) - *H*(***x***)) scores are shown. Other legends are the same as given in Fig. 1.

Table 1 lists the ten lowest- and ten highest (*H*(***x***') - *H*(***x***)) valued probesets and Fig. 2 shows expression profiles for the two lowest- and two highest probesets listed in Table 1. The difference is greatest for the probeset '206319_s_at'. This is mainly because the relative expression for the testis changes from 0.35 to 0.75 by virtue of data processing. ROKU gives a low entropy (*H*(***x***') = 1.950 and *H*(***x***') <*H*(***x***)) for the probeset '206319_s_at' and a high entropy (*H*(***x***') = 4.729 and *H*(***x***') > *H*(***x***)) for the probeset '201131_s_at'. This is quite reasonable because visual evaluation admits the former to be tissue-specific and the latter to be non-specific. Schug's method, however, gives quite similar values (4.235 for the former and 4.228 for the latter) for the two probesets: the entropy for the former is higher than that for the latter.

There are 858 probesets satisfying *H*(***x***') > *H*(***x***): processed expression vectors are less tissue-specific than the original vectors. Visual evaluation for those probesets showed no probeset exists whose entropy score is improperly assigned, i.e., no obvious tissue-specific probesets exist. These results demonstrate the data processing strategy used in ROKU successfully estimated/ranked probesets by their overall tissue specificity on real data. We verified such trends in other microarray datasets (data not shown).

Note that ROKU is inferior to Schug's method (i.e., direct application of entropy to measuring tissue specificity) in rare cases. For example, consider a gene expression pattern of constant high expression in *N*/2 tissues and low expression in other tissues. ROKU gives the processed expression pattern as 'flat' and *H*(***x***') = log_2_(*N*). Accordingly, ROKU cannot distinguish such differential expression patterns from constant expression patterns because it gives the same entropy scores for the two patterns. In other words, *H*(***x***') is not useful for identifying non-specific genes. Nevertheless, this disadvantage is not a problem for detecting the tissue-specific expression patterns we focused on. We also observed that there was no probeset suffer from this disadvantage in the real data set.

### Detection of specific tissues as outliers

As mentioned earlier, the entropy does not indicate which tissues are specific though it can rank genes according to their degrees of overall tissue specificity. To identify such specific tissue when they exist, ROKU employs an outlier-detection-based method proposed by Kadota et al. [3] (see Methods for details). Regardless of over- and/or under-expressed outliers, it can return specific tissues corresponding to outliers for each gene. Accordingly, an outlier matrix can be constructed (consisting of 1 for over-expressed outliers, -1 for under-expressed outliers, and 0 for non-outliers) that corresponds to the original gene expression matrix by applying the method. Genes with any expression pattern of interest can be detected using the outlier matrix. The outlier matrix is also available in the additional file [see Additional file 1].

For example, ROKU identifies 59 probesets specific to lung and 291 probesets specific to fetal lung and of course no redundancies exist between the two sets by virtue of the advantage of the original method [3]. Since ROKU is a combined method consisting of calculation of an entropy and assignment of specific tissues to each gene, ROKU can compensate for the disadvantage of the original method [3] by assigning an entropy score *H*(***x***'): ROKU can rank genes with particular tissue-specific patterns by their overall tissue specificity. We compared the performance of ROKU to that of Schug's *Q*_*t*_(***x***) statistic [4] which can also rank genes specific to a tissue *t*.

**Figure 3:**
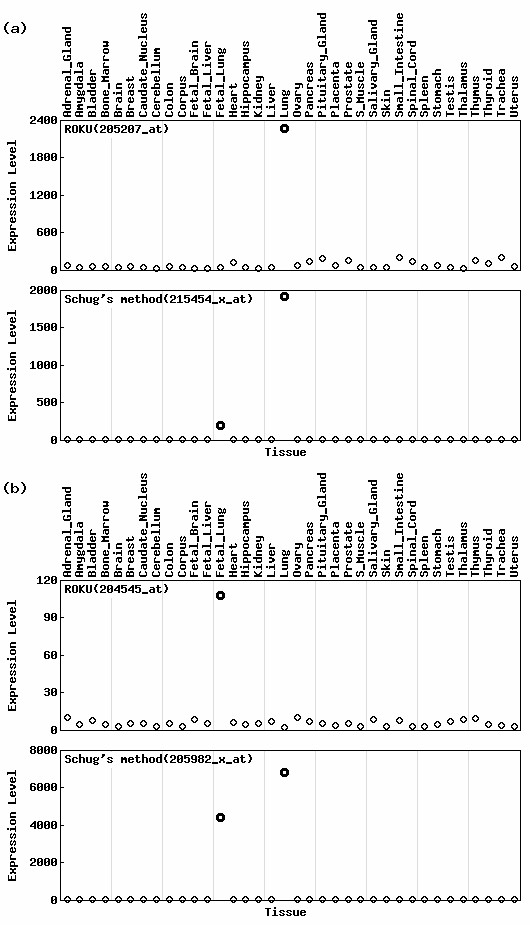
**Expression patterns of probesets listed in Table 2**. Expression patterns of top-ranked probesets specific to (a) lung and to (b) fetal lung are shown. Other legends are the same as given in Fig. 1. Note that the two methods output different top-ranked probesets and probesets detected by ROKU are specific only to the objective tissue.

Fig. 3 shows the top-ranked gene expression profiles specific to (a) lung and (b) fetal lung identified by ROKU's *H*(***x***') statistic and Schug's *Q*_*t*_(***x***) statistic [4]. The *Q*_*t*_(***x***) statistic for a tissue *t *in a gene expression vector ***x ***is defined as *Q*_*t*_(***x***) = *H*(***x***) - log_2_(*p*_*t*_) (see Methods for details). Clearly, ROKU can detect probesets whose expression patterns are specific only to each of the objective tissues (lung or fetal lung) while Schug's *Q *statistic cannot. This is because a low *Q*_*t*_(***x***) statistic indicates that gene ***x ***is relatively highly expressed in a small number of tissues including tissue *t*, but does not always indicate whether the expression pattern of ***x ***is specific only to the tissue *t*. Indeed, both probesets ('215454_x_at' detected as specific to lung and '205982_x_at' specific to fetal lung) identified by Schug's *Q*_*t*_(***x***) statistic include another tissue in addition to the objective tissue. We analyzed this trend in the top-ranking probesets (Table 2). We assert that these probesets are not specific to lung (or fetal lung) but are specific to both lung and fetal lung. Although the choice of which method should be used is, of course, dependent on individual research purposes, our method (ROKU) is superior to Schug's *Q *statistic for detecting genes specific only to tissues of interest.

Of 22,283 probesets analyzed, 16,072 exhibit one or more specific tissues. We observed that most of them consist of specific up-expression patterns, such as Figs. 1c and 1d [see Additional file 1]. This is probably because the distribution of gene expression levels from the dataset we used here roughly follows an exponential distribution in which the probability of a gene's expression observation decays exponentially (data not shown). Still we appreciate the merit of ROKU being able to detect genes with various types of tissue-specific expression patterns, as shown in Fig. 1.

### Effect of different quantification algorithms on gene ranking

**Figure 4:**
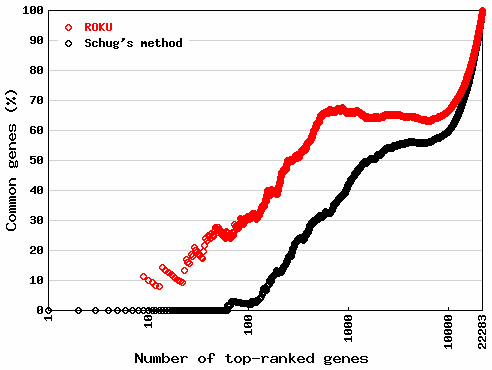
**Effect of different quantification algorithms on gene ranking**. MAS5- and RMA-quantified data are compared. The higher the percentage of common probesets between the two, the more rank-invariant property the method has. ROKU gives a more invariant gene ranking than Schug's method.

As discussed in Grant et al. [6], a serious issue regarding any method is the choice of quantification algorithms, such as MAS5 [7] or RMA [8]; different choices can output different subsets of top-ranked genes. We compared the influence on gene ranking when the same raw data are MAS5-quantified and RMA-quantified. Fig. 4 shows the percentages of common probesets in a top-ranked set of ~22,283 probesets between MAS5 data and RMA data, by gene ranking using ROKU (red circle) and Schug's method (black circle). Although both methods (ROKU and Schug's method) output relatively low percentages of common probesets, especially in the 100 top-ranked probesets (about 31% for ROKU; about 3% for Schug's method), the percentages for ROKU were consistently higher than those for Schug's method. This result indicates gene ranking based on ROKU is more robust against data transformation than Schug's method.

There are some ways for extending this work. First, we used an outlier-detection-based method [3] for the detection of specific tissues. Some other methods, such as Sprent's non-parametric method [5] and its derivative, could be applicable. The outputs of these methods vary with the selected parameters. A comparative study of these methods is the next task. Second, the current work did not discuss the statistical significance of observed differences in gene expression. We plan to combine the significance analysis, such as a method of Sharov et al. [9], with the current method.

## Conclusion

In this work, we propose a novel method (ROKU) for the detection of genes with tissue-specific expression patterns. ROKU was developed to compensate for the disadvantages of two conventional methods [3,4] by combining the advantages of the two. Using synthetic expression data, we demonstrated its potential applicability for the detection of various types of specific expression patterns. Although most of the detected tissue-specific genes in real microarray data exhibit one type of expression pattern (i.e., 'up-type' genes selectively over-expressed in a single or small number of tissues compared to the others), the entropy-based gene ranking by ROKU outperforms the two original methods. ROKU can be a powerful tool for selecting genes specific to tissues of interest.

## Methods

### Microarray data

Publicly available Affymetrix U133A oligonucleotide microarray data for 22,283 genes in 36 various normal human tissues [5] were downloaded from the author's website [10]. For the most part we used the data quantified using MAS5 (Micro Array Suite 5 from Affymetrix) software. Other quantified data using the RMA algorithm [8] were also analyzed to compare the effects of different quantification algorithms. RMA quantification was performed by the justRMA() function in R [11] using raw data (Affymetrix CEL files).

### Gene ranking by Shannon entropy

The use of Shannon entropy [12] to rank genes by their overall tissue specificity here is the same as described in Schug et al. [4]. Consider one gene's expression vector ***x ***= (*x*_*1*_, *x*_*2*_, ..., *x*_*N*_) for *N *tissues and an observation *x*_*t *_for tissue *t*. The entropy of the gene is calculated as

H=−∑t=1Nptlog⁡2(pt),     (1)
 MathType@MTEF@5@5@+=feaafiart1ev1aaatCvAUfKttLearuWrP9MDH5MBPbIqV92AaeXatLxBI9gBaebbnrfifHhDYfgasaacH8akY=wiFfYdH8Gipec8Eeeu0xXdbba9frFj0=OqFfea0dXdd9vqai=hGuQ8kuc9pgc9s8qqaq=dirpe0xb9q8qiLsFr0=vr0=vr0dc8meaabaqaciaacaGaaeqabaqabeGadaaakeaacqWGibascqGH9aqpcqGHsisldaaeWaqaaiabdchaWnaaBaaaleaacqWG0baDaeqaaaqaaiabdsha0jabg2da9iabigdaXaqaaiabd6eaobqdcqGHris5aOGagiiBaWMaei4Ba8Maei4zaC2aaSbaaSqaaiabikdaYaqabaGccqGGOaakcqWGWbaCdaWgaaWcbaGaemiDaqhabeaakiabcMcaPiabcYcaSiaaxMaacaWLjaWaaeWaaeaacqaIXaqmaiaawIcacaGLPaaaaaa@47F0@

where *p*_*t *_is the relative expression of *x*_*t *_for tissue *t *defined as pt=xt/∑t=1Nxt
 MathType@MTEF@5@5@+=feaafiart1ev1aaatCvAUfKttLearuWrP9MDH5MBPbIqV92AaeXatLxBI9gBaebbnrfifHhDYfgasaacH8akY=wiFfYdH8Gipec8Eeeu0xXdbba9frFj0=OqFfea0dXdd9vqai=hGuQ8kuc9pgc9s8qqaq=dirpe0xb9q8qiLsFr0=vr0=vr0dc8meaabaqaciaacaGaaeqabaqabeGadaaakeaacqWGWbaCdaWgaaWcbaGaemiDaqhabeaakiabg2da9iabdIha4naaBaaaleaacqWG0baDaeqaaOGaei4la8YaaabmaeaacqWG4baEdaWgaaWcbaGaemiDaqhabeaaaeaacqWG0baDcqGH9aqpcqaIXaqmaeaacqWGobGta0GaeyyeIuoaaaa@3E61@. *H *ranges from zero to log_2_(*N*), with the value 0 for genes expressed in a single tissue (Fig. 1a) and log_2_(*N*) for genes expressed uniformly in all the interrogated tissues (Fig. 1b).

To equally identify down- and mixed-types of tissue-specific genes as well as up-type genes, we processed the original vector ***x***. The processed observation *x*_*t*_' for tissue *t *is defined as

x′t
 MathType@MTEF@5@5@+=feaafiart1ev1aaatCvAUfKttLearuWrP9MDH5MBPbIqV92AaeXatLxBI9gBaebbnrfifHhDYfgasaacH8akY=wiFfYdH8Gipec8Eeeu0xXdbba9frFj0=OqFfea0dXdd9vqai=hGuQ8kuc9pgc9s8qqaq=dirpe0xb9q8qiLsFr0=vr0=vr0dc8meaabaqaciaacaGaaeqabaqabeGadaaakeaacuWG4baEgaqbamaaBaaaleaacqWG0baDaeqaaaaa@2FCE@ = |*x*_*t *_- *T*_*bw*_|,     (2)

where *T*_*bw *_is the one-step Tukey biweight, a popular statistic robust against outliers. It provides as much robustness as a median and is also used to estimate the expression signal from each probe set in the Affymetrix Micro Array Suite (MAS 5.0) software package [7,13]. The parameters for the calculation of *T*_*bw *_are the same as those adopted in the tukey.biweight() function in R package 'affy' (i.e., *c *= 5, ε = 0.0001) [11]. Our method (ROKU) uses the processed expression vector ***x***' of a gene, while Schug et al. [4] uses the original vector ***x***, to calculate the gene's entropy (*H*(***x***') and *H*(***x***)) as a measure of the overall tissue specificity.

### Detecting specific tissues as outliers

As mentioned above, the entropy does not indicate which tissues are specific, but is a measure of the overall tissue specificity of a gene. We imagine observations in specific tissues to be easily visualized as outliers on the over- and/or under-expressed side if any exist. We used an outlier-detection-based method proposed by Kadota et al. [3] to detect tissues with specific expression patterns. According to Kadota et al. [3], the statistic *U *for identifying outliers is defined as

U=nlog⁡σ+2×s×log⁡n!n,     (3)
 MathType@MTEF@5@5@+=feaafiart1ev1aaatCvAUfKttLearuWrP9MDH5MBPbIqV92AaeXatLxBI9gBaebbnrfifHhDYfgasaacH8akY=wiFfYdH8Gipec8Eeeu0xXdbba9frFj0=OqFfea0dXdd9vqai=hGuQ8kuc9pgc9s8qqaq=dirpe0xb9q8qiLsFr0=vr0=vr0dc8meaabaqaciaacaGaaeqabaqabeGadaaakeaacqWGvbqvcqGH9aqpcqWGUbGBcyGGSbaBcqGGVbWBcqGGNbWzcqaHdpWCcqGHRaWkdaGcaaqaaiabikdaYaWcbeaakiabgEna0kabdohaZjabgEna0oaalaaabaGagiiBaWMaei4Ba8Maei4zaCMaemOBa4MaeiyiaecabaGaemOBa4gaaiabcYcaSiaaxMaacaWLjaWaaeWaaeaacqaIZaWmaiaawIcacaGLPaaaaaa@4A24@

where *n *and *s *denote the numbers of non-outlier and outlier candidates, and σ denotes the standard deviation (SD) of the observations of the *n *non-outlier candidates. The procedure is first, normalize the gene vector ***x ***= (*x*_1_, *x*_2_, ..., *x*_*N*_) for *N *(=*n*+*s*) tissues by subtracting the mean and dividing by the SD; second, sort the normalized values (i.e., *Z*-scores) by order; third, calculate the statistics *U *for various combinations of outlier candidates starting from both sides of the values; finally, regard tissues corresponding to outliers detected in the combination of the minimum *U *as 'specific'.

## Authors' contributions

KK invented the method and wrote the paper. JY made critical comments in light of the current algorithm. YN, TT, and KS provided critical comments and led the project.

## Supplementary Material

Additional file 1 **Full information analyzed by ROKU for dataset of Ge et al. (2005)**. For the original gene expression matrix, an outlier matrix (consisting of 1 for over-expressed outliers, -1 for under-expressed outliers, and 0 for non-outliers) is provided. It also contains two entropy scores measured by ROKU and Schug's method and their ranks. Click here for file

## Figures and Tables

**Table 1:** Comparison of entropy scores by ROKU and Schug's method. Probesets and two entropy scores estimated by ROKU and Schug's method are shown. The numbers in parenthesis are the ranks of the probesets. One can easily rank all probesets by this order using the additional file [see Additional file 1].

Probeset ID	Entropy		*H*(***x***') - *H*(***x***)
		
	*H*(***x***')	*H*(***x***)	
*Probesets with the lowest (H(***x***') - H(***x***)) scores*			
206319_s_at	1.950 (482)	4.235 (2347)	-2.285
215622_x_at	1.499 (295)	3.698 (1144)	-2.198
207636_at	1.253 (210)	3.369 (806)	-2.116
206587_at	2.310 (651)	4.402 (3048)	-2.092
206945_at	1.659 (352)	3.742 (1222)	-2.083
213332_at	2.674 (861)	4.755 (6637)	-2.081
215189_at	1.706 (371)	3.784 (1292)	-2.078
207053_at	2.321 (653)	4.359 (2830)	-2.038
207908_at	1.420 (260)	3.452 (877)	-2.032
214377_s_at	0.808 (105)	2.830 (493)	-2.022
			
*Probesets with the highest (H(***x***') - H(***x***)) scores*			
202546_at	4.955 (22257)	4.431 (3215)	0.524
201131_s_at	4.729 (17433)	4.228 (2311)	0.501
211981_at	4.746 (18161)	4.328 (2680)	0.419
202864_s_at	4.919 (22137)	4.502 (3668)	0.417
213998_s_at	4.652 (14417)	4.259 (2432)	0.393
218839_at	4.971 (22267)	4.580 (4284)	0.391
208963_x_at	4.312 (6836)	3.930 (1548)	0.382
210474_s_at	4.985 (22277)	4.622 (4701)	0.363
213071_at	4.551 (11124)	4.196 (2200)	0.355
218361_at	4.944 (22231)	4.617 (4642)	0.326

**Table 2:** List of top ten genes specific to lung and to fetal lung. Probesets and two entropy scores estimated by ROKU and Schug's method are shown. *Probesets indicate those listed to be "specific to lung" are also listed to be "specific to fetal lung" and vice versa. Note that Schug's method has strong redundancy when similar tissues are selected independently and therefore cannot detect probesets specific only to the objective tissue.

ROKU		Schug's method	
Probeset ID	*H*(***x***')	Probeset ID	*Q*(***x***)

*Specific to Lung*			
205207_at	2.483	215454_x_at *	1.223
215677_s_at	4.006	218835_at	1.310
206432_at	4.020	214199_at	1.512
218627_at	4.066	211735_x_at *	1.941
204622_x_at	4.255	205982_x_at *	1.959
205624_at	4.290	214387_x_at *	1.994
216782_at	4.339	37004_at *	2.311
206026_s_at	4.361	209810_at *	2.521
219361_s_at	4.378	217046_s_at	2.820
205027_s_at	4.414	205819_at	3.498
			
*Specific to Fetal lung*			
204545_at	2.779	205982_x_at *	2.591
221418_s_at	2.879	211735_x_at *	2.662
206315_at	3.252	214387_x_at *	2.863
211300_s_at	3.369	37004_at *	3.151
213417_at	3.399	209810_at *	3.258
206159_at	3.791	38691_s_at	3.716
208474_at	3.883	203417_at	4.307
220707_s_at	3.936	211237_s_at	4.480
206646_at	4.039	215454_x_at *	4.494
221284_s_at	4.044	204468_s_at	4.637
